# Time resolved X-ray Dark-Field Tomography Revealing Water Transport in a Fresh Cement Sample

**DOI:** 10.1038/srep29108

**Published:** 2016-06-30

**Authors:** Friedrich Prade, Kai Fischer, Detlef Heinz, Pascal Meyer, Jürgen Mohr, Franz Pfeiffer

**Affiliations:** 1 Lehrstuhl für Biomedizinische Physik, Physik-Department & Institut für Medizintechnik, Technische Universität München, Garching, 85748, Germany; 2 Centrum Baustoffe und Materialprufung, Technische Universität München, München, 81245, Germany; 3 Institut für Mikrostrukturtechnik, Karlsruher Institut für Technologie, 76344, Eggenstein-Leopoldshafen, Germany; 4 Institut für diagnostische und interventionelle Radiologie, Klinikum rechts der Isar, Technische Universität München, 81675, München, Germany

## Abstract

Grating-based X-ray dark-field tomography is a promising technique for biomedical and materials research. Even if the resolution of conventional X-ray tomography does not suffice to resolve relevant structures, the dark-field signal provides valuable information about the sub-pixel microstructural properties of the sample. Here, we report on the potential of X-ray dark-field imaging to be used for time-resolved three-dimensional studies. By repeating consecutive tomography scans on a fresh cement sample, we were able to study the hardening dynamics of the cement paste in three dimensions over time. The hardening of the cement was accompanied by a strong decrease in the dark-field signal pointing to microstructural changes within the cement paste. Furthermore our results hint at the transport of water from certain limestone grains, which were embedded in the sample, to the cement paste during the process of hardening. This is indicated by an increasing scattering signal which was observed for two of the six tested limestone grains. Electron microscopy images revealed a distinct porous structure only for those two grains which supports the following interpretation of our results. When the water filled pores of the limestone grains empty during the experiment the scattering signal of the grains increases.

X-ray imaging has developed into a valuable tool for many research areas over the last decades. Besides it’s common use in clinical diagnostics, X-ray imaging is also often applied in biomedical and materials research. Here, it enables scientists to study the internal structural properties of a sample in a non-destructive manner^1^. Especially, since the macroscopic mechanical properties of a material are often determined by it’s underlying microstructure, a detailed knowledge on this micostructure is crucial for the development of reliable and sustainable materials. Polymer-based composite^2^,^3^ and cementitious materials^4^,^5^ are just two examples of many materials which are intensively studied with X-ray computed tomography (CT).

Recent advances show promise to even increase the value of X-ray imaging for such applications. One of these advances is the development of X-ray Talbot-Lau interferometry^6^. Besides the standard attenuation contrast, this technique readily provides two additional contrast modes at laboratory-based imaging setups utilizing interferometric effects from grating structures. The differential phase-contrast^6^,^7^ originates from the refraction of X-rays and it can provide improved soft-tissue contrast in biomedical research when compared to the attenuation contrast^8^. The second additional contrast is often referred to as the dark-field contrast^9^ and it is sensitive to sub-pixel features within the sample. It originates from small-angle X-ray scattering^10^ and therefore carries quantitative information on microstructures that can not be directly resolved by the imaging system^11^,^12^. Further effects have also been studied for their contribution to the dark-field signal such as beam hardening, sharp edges within the sample and lens effects^13^,^14^,^15^. However, in the present study we will only consider scattering as the major contribution to the measured signal due to the morphology of the sample under investigation. Thus, we will also refer to the dark-field contrast as the scattering signal.

Grating-based X-ray dark-field imaging has attracted interest in materials research in recent years. The orientation-selective sensitivity of the dark-field signal could be utilized in order to study the orientation of microstructures in anisotropic materials such as bones^16^ and fiber-reinforced polymers^17^,^18^. In another exemplary application of the dark-field signal, the pattern of sub-pixel microcracks was successfully analyzed^19^. Cementitious materials are a further class of materials that has been extensively studied based on X-ray phase-contrast and dark-field imaging. For example, the water transport in porous materials such as mortar could successfully be studied by dark-field imaging^20^. Further work showed that an improved segmentation of the aggregates and the matrix material could be obtained by a phase-contrast CT of a mortar sample, while a dark-field CT revealed microcracks within the aggregates^21^. The matrix of a cementitious material is called cement paste and it initially consists of small particles suspended in water. Silicon dioxide, calcium, alumina and ferric oxide are the main chemical constituents of these particles while their diameter can reach from 0.1 up to 100 *μ*m, thus, giving rise to a strong scattering signal. The essential mechanism which hardens the cement paste is the hydration of these compounds leading to an interconnected network of hydration products. When studying this reaction a very distinct drop of the scattering signal was observed in dark-field images acquired throughout the process of hardening^22^. This finding offers the possibility to study local deviations from the hydration dynamics of the cement paste, something that is impossible with conventional testing techniques which only provide bulk information on the hydration dynamics in large samples^23^.

In this work, we present a method which extends the time-resolved two-dimensional dark-field studies on cementitious materials by the third dimension. We make use of the fact that the dark-field signal of isotropically scattering materials is also compatible with CT^24^,^25^. By acquiring several consecutive dark-field CTs of a fresh cement sample also containing some limestone grains, we show that the hydration dynamics within the cement paste can even be studied in three dimensions over the course of 37 hours.

Furthermore we were able to study the behavior of the limestone grains embedded in the cement paste during the experiment. Our results indicate that water is transported from limestone grains to the cement paste under certain conditions. This internal water supply to the cementitious material during hydration can be referred to as internal curing, a method which is of extreme interest for cement research. Our results are further supported by polarized light microscopy (PLM) and scanning electron microscopy (SEM) experiments revealing a distinct pore system for the affected grains.

## Results

As mentioned in the introduction, the impact of the cement’s hydration on the dark-field signal was previously studied with succeeding dark-field images acquired during the hardening of cement^22^. The observed decline of the cement’s scattering signal is related to the growth of the hydrate phase and to the shrinkage of the initial cement particles^22^. Contrary to this, we observed an increase of the scattering signal when aggregates such as limestone grains were added to the cement paste in further experiments. Here, we report on these effects based on a time-resolved three-dimensional dark-field CT study.

**Figure 1:**
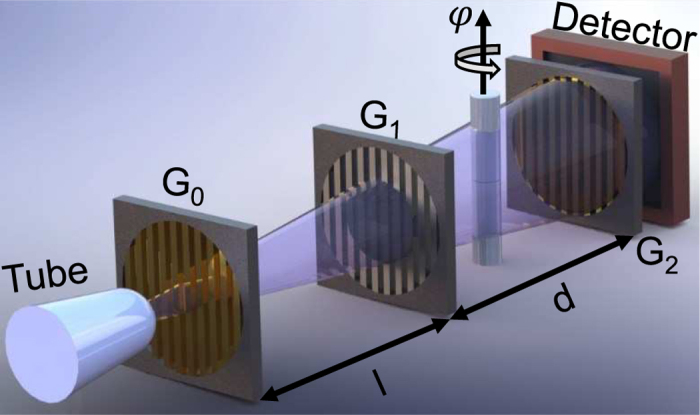
The experimental setup consisting of (from left to right) the X-ray tube, the source grating *G*_0_, the phase grating *G*_1_, the sample (cylinder) and the tomographic rotation axis (indicated by the black arrow pointing upwards), the analyzer grating *G*_2_ and the X-ray detector. The inter grating distances *l* and *d* were both 92.5 cm. Further details on the setup can be found in ref. 12. During a CT scan the sample is rotated around the tomographic axis while images are acquired for several angles *φ*.

The experimental setup used in this study is schematically shown in Fig. 1. The cylindrical plastic tube containing the cement paste and the limestone grains of three different types was placed between the phase- and the analyzer grating. It was rotated around the tomographic axes indicated by the arrow on top of the sample and transmission and dark-field images were acquired for several angles *φ* = 0° … 360°.

**Figure 2:**
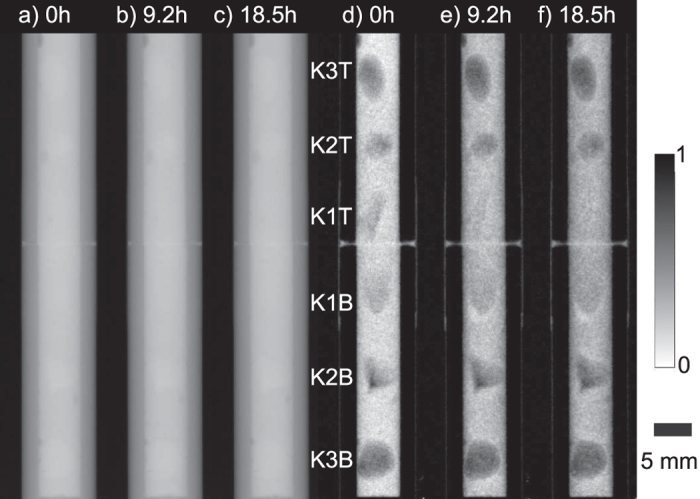
Transmission (**a**–**c**) and dark-field (**d**,**e**) images taken after 0 (**a**,**d**), 9.2 (**b**,**e**) and 18.5 hours (**c**,**f**) under the same projection angle *φ* = 0. While the transmission images do not exhibit any change, the dark-field signal of the cement and the two limestone grains of type 1 clearly changes over time.

Figure 2 shows some transmission (a–c) and dark-field images (d–f) for the angle *φ* = 0° at different points in time. Here, the above mentioned effects can clearly be seen. While the sample remains unchanged in the transmission images, a strong change is observed in the dark-field images. At *t* = 0 hours the six limestone grains are clearly visible in the dark-field image and their approximate vertical position is marked in Fig. 2. While a clear contrast between the limestone grains of type 2 and 3 (i.e. the grains *K*2*T*, *K*2*B* and *K*3*T*, *K*3*B* respectively) and the cement paste remains in the dark-field images during time, this contrast diminishes with time for the limestone grains of type 1 (i.e. the grains *K*1*T*, *K*1*B*). However, the two-dimensional images shown in Fig. 2 are insufficient in order to localize this effect. In order to clarify whether this effect is taking place inside the limestone grains of type 1 or within the cement paste surrounding these grains, a new method was required that allowed us to study the internal features of the sample in three dimensions during the course of the experiment.

We achieved this by successively performing 8 dark-field CTs on the sample over 37 hours. In a standard CT measurement, images of the sample are acquired for many angles *φ* = 0° … 360°. From these two dimensional images the attenuation and scattering coefficients *μ* and *ε* of the sample are reconstructed in three dimensions. The relations between the dark-field signal and *ε* and the transmission signal and *μ* are stated in the methods section. For a sample that changes during the acquisition of one CT scan like it is the case for hardening cement paste, such a reconstruction represents a time average of *μ* and *ε* averaged over the duration of one CT scan. So the initial time resolution of our experiments was 4.6 hours as we would get one reconstruction for *μ* and *ε* from each CT scan. In order to improve the time resolution, we computed more than 8 reconstructions using a continuously shifted subset of all acquired images for one reconstruction. A detailed description on this procedure is given in the methods section. As a result the time frame over which *μ* and *ε* get averaged is shifted as well. Thus, we were able to compute 147 reconstructions of the sample providing us with a virtual time resolution of 13.4 minutes.

**Figure 3:**
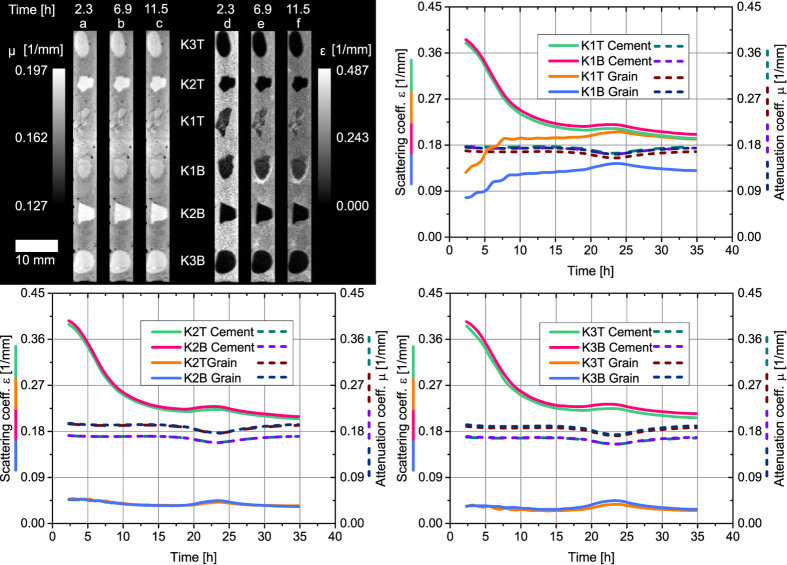
The reconstructed attenuation coefficient *μ* (**a**–**c**) and scattering coefficient *ε* (**d**–**f**) of a slice through the center of the sample is shown after 2.3 (**a**,**d**), 6.9 (**b**,**e**) and 11.5 hours (**c**,**f**). A change of *ε* is observed in the cement and the limestone grains of type 1. The temporal evolution of *μ* and *ε* for the cement and each grain throughout the whole experiment is depicted in the three graphs. The scattering coefficient of the cement paste in the vicinity of each grain shows a clear logistic decline over time (pink and green curve in each individual graph) which has been reported before^22^. In contrast to *K*2*T*, *K*3*T*, *K*2*B* and *K*3*B*, *ε* increases for the two limestone grains *K*1*T* and *K*1*B* (light-blue and orange curve in each individual graph). The attenuation coefficient remains constant for the cement paste and each limestone grain (dashed curves in each individual graph). The small dip and bump in the signals between 20–25 hours is related to an instability in the high voltage generator of the tube.

Figure 3 shows the attenuation (a–c) and scattering coefficient *μ* and *ε* (d–f) from three of these reconstructions for a slice through the center of the sample. The plastic tube was masked out in these images. A movie showing the same slice from all 147 reconstructions is provided in the supplementary information. Similar as for the images in Fig. 2, the appearance of the sample does not change when comparing the reconstructed slices of the attenuation coefficient. The reconstructed slices of the scattering coefficient in Fig. 3, however, clearly show how the cement’s *ε* decreases with time. This effect takes place throughout the whole sample, agreeing with the observations from the dark-field images in Fig. 2. Focusing on the limestone grains, one can now identify that the grains of type 1 are responsible for the effect that was already observed in the images of Fig. 2. While *ε* remains close to zero within the grains of type 2 and 3, an increase of *ε* is observed in the grains of type 1. This increase can clearly be detected for grain *K*1*T*, while it is slightly smaller for grain *K*1*B*. Based on a segmentation of the cement paste and the cement grains, we extracted average values for *μ* and *ε* for the cement surrounding each grain as well as for each single grain. The graphs in Fig. 3 show the result of this procedure, which was performed on each of the 147 reconstructions. A movie visualizing the segmentation process can be found in the supplementary information.

The grains of type 2 and 3 have a slightly higher attenuation coefficient when compared to the cement paste as is shown by the dashed curves. In contrast to that, the type-1 grains have a similar *μ*-value as the cement indicating that they are less dense when compared to the type-2 and type-3 grains. The decrease of *ε* within the cement paste in the vicinity of each grain is shown by the green and pink solid curve in each graph. The observed logistic shape has already been reported and is further discussed in ref. 22.

The progression of the averaged scattering coefficient of the limestone grains is depicted by the blue and orange solid curve in each graph. As expected *ε* strongly increases for grain *K*1*T*, while the increase is slightly less for grain *K*1*B*. In contrast to that, the four grains of type 2 and 3 show a much smaller, constant scattering coefficient and no increasing trend is observed here.

**Figure 4:**
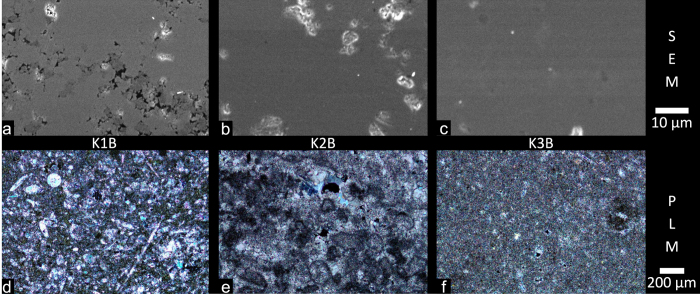
Scanning electron microscopy (SEM) (**a**–**c**) and polarized light microscopy (PLM) (**d**–**f**) images for the samples *K*1*B* (**a**,**d**), *K*2*B* (**b**,**e**) and *K*3*B* (**c**,**f**). The limestone grain *K*1*B* shows a clear porous structure (see panel a), while the other grains do not show a significant porosity in the SEM images (see panel b and c). The bright spots observed especially in *K*2*B* correspond to surface charging of isolated regions within the grain. The PLM images show that all grains are made of pure calcite while *K*3 also contains some quartz particles. *K*1 contains a lot of fossil fragments (panel d) embedded in a microcrystalline (micritic) carbonate matrix. *K*2 (panel e) is a fine crystalline to micritic limestone with round elements (spherulites). In *K*3 (panel f), single areas of fine crystalline calcite are found in an otherwise majorly micritic matrix. More SEM and PLM images can be found in Figs S1 and S2 of the supplementary information.

For a better understanding of these observations, we carried out microscopy experiments on a thin section of the sample, which was prepared after the CT measurements. The results of the SEM and PLM experiments are depicted in Fig. 4 in panels a–c and d–f respectively. Here, only images of the grains *K*1*B*, *K*2*B* and *K*3*B* are shown, while images for the other grains are shown in Fig. S1 in the supplementary information. The three limestone grains can be characterized in terms of their chemical composition as well as of their crystalline structure based on the PLM images. All grains can clearly be classified as pure calcite except for the type-3 grain which also contains some small isolated quartz particles. Furthermore, fine crystalline fossil fragments embedded in a carbonate matrix are found in the type-1 grains. The matrix is majorly of micritic appearance meaning that the single crystals are smaller than 63 *μ*m in size. The grains of type 2 consist of fine crystalline regions alongside micritic regions. Circular features, so called spherulites, with a very fine and dense calcite border are found in the type-2 grains. The limestone of type 3 shows a purely micritic structure, only single isolated fine crystalline regions are found in this type. These characteristics support the findings of the dark-field CT from a chemical point of view. Because the grains purely consist of calcite, no scattering contrast between different materials exists while the cement is much more heterogeneous giving rise too a strong scattering contrast between the different materials. Therefore the scattering coefficients observed for all grains are very small when compared to the cement’s scattering coefficient. From these findings, however, a distinction between the different types of limestone which explains the observed effects is not obvious. Thus, we further acquired SEM images. The type-1 limestone can clearly be distinguished from the other two types in the SEM images in Fig. 4 as it exhibits a very distinct microporous system. First of all this explains the density difference of the three limestone types which we observed in the attenuation CT data. Secondly the impact of these micropores on the dark-field results becomes clear in the context of a recent publication^20^. It was shown that the intrusion of water into a porous material results in a decrease of the scattering signal of this material. This process must be reversed in our case since the scattering signal of the type-1 limestone grains increases throughout the experiment. Thus, the initially water filled pores most likely emptied during the hydration of the surrounding cement paste. Because the scattering contrast between water and calcite is less when compared to the scattering contrast between air and calcite, the emptying of pores results in an increase of the scattering signal of the type-1 limestone. The limestone grains were added in a dry state to the cement paste during mixing. Thus, the pores in the type-1 limestone initially saturated with water during mixing and finally the water was drawn out of the grains into the cement paste due to capillary forces. These capillary forces are caused by the formation of pores within the cement paste due to the hydration of the cementitious material^26^.

## Discussion

Our results show the feasibility of applying grating-based dark-field CT in order to study slow processes in three dimensions as well as resolved in time. However, we emphasize that the time resolution of the presented technique of course is not sufficient in order to observe swift changes in the sample taking place on the scale of a few minutes. Therefore it is necessary to decrease the duration of one single CT scan, which for example could be achieved by advanced acquisition schemes^27^,^28^. The duration of one CT could already be decreased in test studies down to one hour by simply acquiring less projections, decreasing the exposure time and the spatial resolution of the scan. The latter point is quite crucial for dark-field studies, since the origin of the signal allows one to study processes on a length scale which is well below the spatial resolution of the imaging system. As shown in this study, we were able to visualize the transport of water from pores with a size of a few micrometers while the spatial resolution was only 93 *μ*m. This aspect and the fact that time-resolved tomography becomes feasible with shorter measurement time makes grating-based X-ray dark-field imaging extremely attractive for the study of slow dynamic processes on the micrometer length-scale.

As an application example we showed how water transport in cementitious materials can be studied with this method. During our experiment the cement paste was supplied with additional water from the inside by the limestone. This observed effect can be understood as an internal curing experiment^29^. In contrast to that, external curing is referred to a method where additional water is supplied from the outside. Since this water supply affects the mechanical properties as well as the long term durability of cement, internal curing is an active field in cement research. Therefore the presented work is of extreme interest to researchers working in that field.

## Methods

### Sample Preparation

The cement paste was prepared according to the same protocol used in ref. 22. The same type of cement (CEM I 42.5 R) was used with a water to cement ratio of 0.4. Two grains from three different batches (type 1, 2, 3) of limestone were chosen. These grains were separately mixed with the cement and the two grains of each batch were separately placed into two plastic tubes along with some cement paste. The plastic tubes had an outer diameter of 1cm and an inner diameter of 0.6 cm. The two tubes were stacked with the opening facing each other and they were fixed together with a few windings of adhesive tape. The sample was mounted onto the tomographic stage of the grating interferometer and the measurement was started approximately 15 minutes after the initial contact of the cement powder with water.

### Dark-field Tomography

A detailed description on the interferometer used in this study can be found in ref. 12. The X-ray tube was operated at 60 kVp with a target power of 70 W. A mean visibility of 22.5 percent was measured in the central region of the field of view before the measurement. The sample was placed between the phase and the analyzer grating with a distance of 42 cm to the phase grating. Thus an effective pixel size of 93 *μ*m was achieved. Eight full 360 degree rotations of the sample were performed over 37 hours, each of them consisting of *n* = 441 angular positions for *φ*. In total *N* = 3528 angular projections were acquired. Each full rotation was divided into 21 blocks with each block consisting of 5 reference scans (without sample) and 21 sample scans for different angles *φ*. For each reference and sample scan, 7 phase-steps^7^ were acquired with an exposure time of 2 seconds per step. In addition to the rotation, the sample was also shifted by a random but logged number of pixels in horizontal direction for each angular sample scan in order to avoid ring artifacts in the reconstruction.More information on this method for ring artifact reduction is given in the supplementary information for this article and ref. 30.

### Processing, Reconstruction and Data Analysis

Each sample scan was processed with the averaged data of the 5 previously acquired reference scans. We only processed the transmission and dark-field images since the differential phase-contrast images did not provide any meaningful information as it already is described in ref. 22.

The transmission (*T*) and dark-field (*D*) signals are in general related to the attenuation and scattering coefficient *μ* and *ε* of the sample by the Beer-Lambert law^24^,^25^.


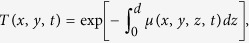



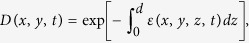


Here, *x* and *y* mark the horizontal and vertical pixel position on the detector while *z* corresponds to the axis in direction of the X-ray beam. *μ* and *ε* are integrated over the whole thickness *d* of the sample. The dependence on the time *t* indicates that *μ* and *ε* change over time. It is important to mention that in this representation *μ* and *ε* also contain information on the measurement geometry as well as the characteristics of the gratings. Thus, a quantitative comparison between different interferometer setups is not straight forward. However, this does not influence the qualitative observations made in this study. From the two-dimensional images *T*(*x*, *y*) and *D*(*x*, *y*) acquired at angles *φ* = 0° … 360° the three dimensional distribution of *μ*(*x*, *y*, *z*) and *ε*(*x*, *y*, *z*) can be reconstructed.

The obtained images were shifted back to the initial horizontal sample position with the help of the logged shifts. We applied a simple filtered back projection for the reconstruction. In order to virtually increase the temporal resolution of our measurement, we sorted the images in such a way that more than 8 reconstructions of the sample could be computed. As the total measurement time was 37 hours and 8 minutes we decided to compute *v* = 147 reconstructions providing a time resolution of 13.4 minutes for each reconstruction. In order to achieve this, a reconstruction was performed on the images i * *N* − *n*)/*v* to *i* * (*N* − *n*)/*v* + *n* with *i* = 0, 1,..., *v*. These image subsets were further sorted so that the first image in each subset corresponded to *φ* = 0 in order to assure that the reconstructions were intrinsically registered.

For data analysis, the first reconstruction of the scattering coefficient *ε* was used in order to segment each limestone grain from the cement paste. We used the commercially available software VGStudio MAX 2.1 from Volume Graphics GmbH, Germany for the segmentation. Based on this segmentation, we extracted binary pixel masks labeling the pixels which corresponded to the limestone grains *K*1*T*, *K*2*T*, *K*3*T*, *K*1*B*, *K*2*B*, *K*3*B* and the surrounding cement paste. The mean scattering coefficient *ε* and the mean attenuation coefficient *μ* was extracted from each reconstruction for the cement paste and the six limestone grains using these masks. Thus, the time evolution of the two signals *μ* and *ε* were obtained. The values from each reconstruction were assigned to the time after which the half of the corresponding image subset was acquired during the measurement. Therefore the first and last data point was assigned to a time of *t* = 2.3 and *t* = 34.8 hours, respectively.

### Polarized Light Microscopy

A thin section through the central region of the sample was prepared for the PLM experiment with a thickness of 20 *μ*m. In order to avoid the formation of a relief due to the cutting process, the sample was diamond polished. The sample was further vacuum impregnated in a fluorescent resin. The experiments were carried out with an Olympus BX 61 light microscope using a crossed polarizer.

### Electron Microscopy

After the PLM experiment, we used the same thin section of the sample for the SEM experiment. In order to lower surface charging of the sample, it was sputtered with carbon prior to the SEM experiment. We used a JEOL-JSM-6060LV scanning electron microscope from Jeol, Eching, Germany with an acceleration voltage of 10 kV. Several images of each grain were collected with a back-scattered electron detector at different magnification factors reaching from 15 up to 2000. The SEM images of Fig. 4 in the main text and Fig. S1 of the supplementary information were acquired with a magnification of 2000 while the SEM images in Fig. S2 of the supplementary information show the grains with a magnification of 200.

## Additional Information

**How to cite this article**: Prade, F. *et al*. Time resolved X-ray Dark-Field Tomography Revealing Water Transport in a Fresh Cement Sample. *Sci. Rep.* **6**, 29108; doi: 10.1038/srep29108 (2016).

## Supplementary Material

Supplementary Information

Supplementary Information 1

Supplementary Information File
