# Supplementary material for: Time resolved X-ray Dark-Field Tomography Revealing Water Transport in a Fresh Cement Sample
Source: Sci Rep. 2016 Jun 30;6:29108. doi: 10.1038/srep29108 (PMC4928120; doi:10.1038/srep29108)
Supplement: Supplementary Information [file srep29108-s1.pdf]

Supplementary Information: Time resolved X-ray Dark-Field Tomography Revealing Water Transport in a Fresh Cement Sample

Friedrich Prade[1,*], Kai Fischer[2], Detlef Heinz[2], Pascal Meyer[3], Jürgen Mohr[3], and Franz Pfeiffer[1,4]

[1]Lehrstuhl für Biomedizinische Physik, Physik-Department & Institut für Medizintechnik, Technische Universität München, Garching, 85748, Germany
[2]Centrum Baustoffe und Materialprufung, Technische Universität München, München, 81245, Germany
[3]Institut für Mikrostrukturtechnik, Karlsruher Institut für Technologie, 76344, Eggenstein-Leopoldshafen, Germany
[4]Institut für diagnostische und interventionelle Radiologie, Klinikum rechts der Isar, Technische Universität München, 81675, München, Germany
[*]friedrich.prade@ph.tum.de

SEM and PLM images

Coming from the same type of limestone as the grains $K1B, K2B$ and $K3B$, respectively, they have very similar characteristics when compared to figure 4 in the main text. The limestone of type 1 shows a clear pore system while type 2 and 3 do not show any sings of a significant porosity. The crystalline structures of the grains $K1T, K2T$ and $K3T$ is in good agreement with the structure of the grains $K1B, K2B$ and $K3B$ when comparing the PLM images in this figure with the PLM images of figure 4 in the main text.

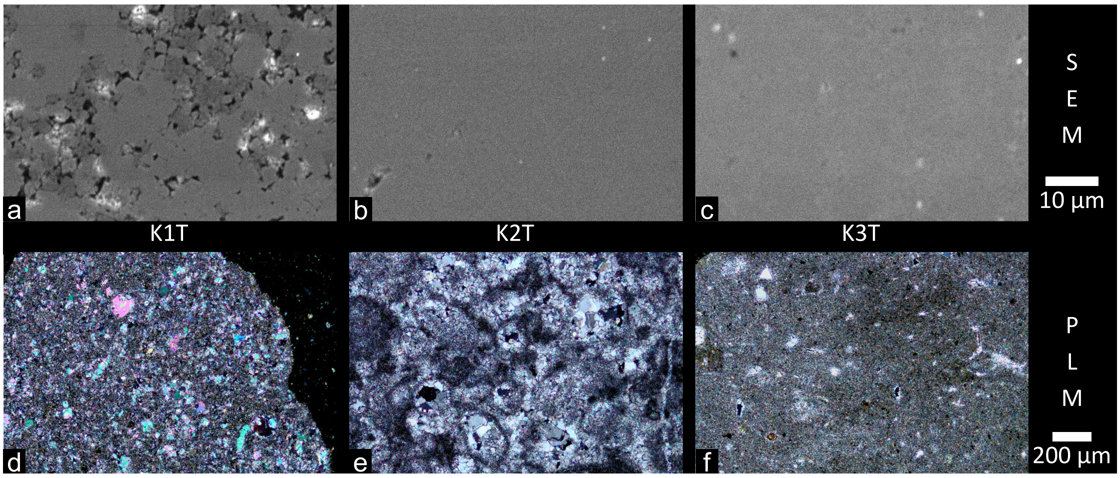

Figure S1. SEM and PLM images for the limestone grains $K1T, K2T$ and $K3T$.

SEM images at smaller magnification

The porosity for the type-1 limestone is homogeneously distributed over the region of interest (panel a and d). In contrast to that no significant porosity is observed for the other two limestone types even with this enlarged region of interest (panel b, c, e and f).

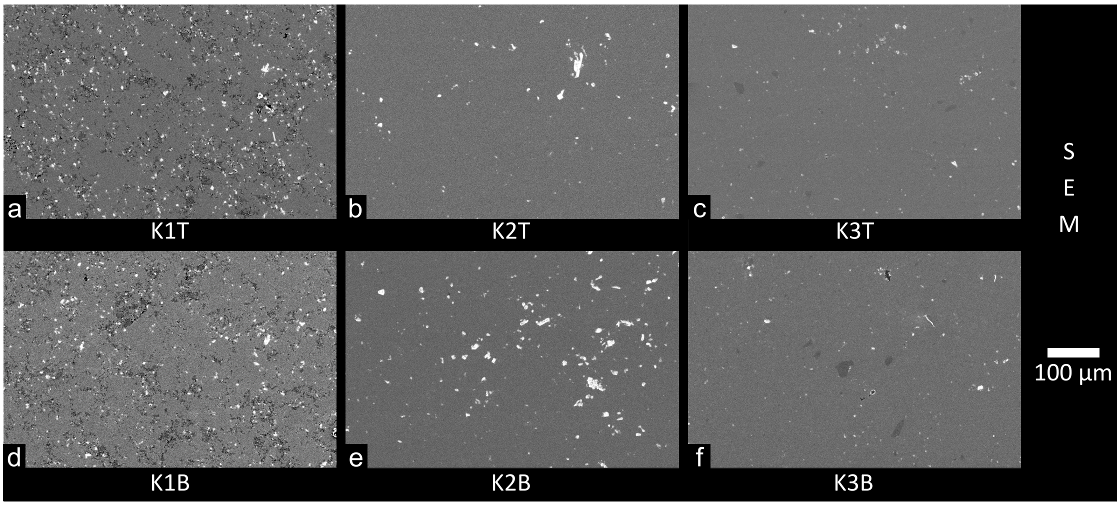

Figure S2. SEM images of all six grains with an magnification of showing a larger region of interest within the grains.

Movies attached to this article

As mentioned in the main text a movie is attached to this article with the filename ”AttScat.avi”. It shows the same slice that is shown in figure 3 of the main text for all of the 147 volumes. Furthermore the file ”RenderedMovie.avi” shows a rendered volume of the attenuation and scattering coefficient at $t = 2.3$ hours. It also shows how the grains are segmented from the cement and finally an overlay of the movie from the file ”AttScat.avi” with the rendered volumes of the attenuation data.

Ring Artifact Reduction

Ring artifacts are a common phenomenon in computed tomography which corrupt the reconstructed images by strong ring structures. They are caused by inhomogeneous responses of the detector pixels. In commercially available MicroCT systems a random detector shift during image acquisition is implemented as an effective hardware based technique to reduce these ring artifacts. By doing so the contribution of the corrupted pixels is averaged over a larger region in the reconstruction and the ring artifacts are greatly reduced. Information on this can be found for example in the publication of Y. Zhu, Medical Physics, 40, 2013, “Micro-CT artifacts reduction based on detector random shifting and fast data inpainting”. Since the detector cannot be moved in our experimental setup we compensate for the ring artifacts by a random shift of the sample. This is feasible for small magnification factors as it is the case for the presented study. It gives the same ring artifact reduction as the commonly done detector shift and the same considerations apply such as an increase in scan time and a reduction in resolution. However the latter being negligible since the presented study does not require high resolution anyway.
